# Dr. Bryce and the Virginia Medical College

**Published:** 1887-09

**Authors:** 


### DR. BRYCE AND THE VIRGINIA MEDICAL COLLEGE.

In a recent number of the *Southern Clinic*, edited by C. A. Bryce, M. D., of Richmond, Virginia, we notice that a somewhat bitter controversy has waged between the editor and some party writing through the *Labor Herald*, a newspaper.

We are sorry to note that it has assumed such bitter personality, which, however strong the provocation between the parties, embarrasses the expression of opinion by disinterested friends and impartial readers.

If charges are fairly made and camly responded to, no one desiring to give impartial criticism need be embarrassed, and no harm will result if a simple misunderstanding or mistake has been made, as then the entire public will see who is in the right, and the wrong party can and ought to make the *amende honorable*, and an amicable adjustment follow. Of this we find an honorable example in the closing paragraph of Dr. O. F. Mason to Dr. Bryce, wherein parties once alienated resumed their former friendship after a full and true understanding of the facts.

Possibly the same adjustment might have followed in the present controversy between Dr. Bryce and a supposed member of the Faculty of the Virginia Medical College had the charges been fairly and calmly presented, through a professional medium and in a manner not to provoke a harsh rejoinder. If this had been the case, friends would not be embarrassed in expressing a free and full opinion, and in approaching the parties with the view to an honorable adjustment. It is no evidence of want of friendship to point out to another plainly and positively his wrong, and the party thus admonished will rarely fail to receive and act upon the impartial suggestions of friends.

Could we certainly know, in the light of every fact who is really at fault, we would not hesitate to say so, not fearing that any true Virginian would take exception thereto.

In the present case Dr. Bryce was assaulted by an anonymous writer in the *Labor Herald* with the charge of gross ingratitude, in the fact that he had through the *Southern Clinic*, his medical journal, severely criticised the Virginia Medical College, after failing to pay his tuition fees in said institution.

As a journalist, we hold that Dr. Bryce, or any other journalist, is justifiable in exposing wrongs committed by a public educational institution when the charges of such wrongs are founded on truth, and when they are in violation of the laws that control such institutions and the honor of the profession. Of course we do not assume to know that Dr. Bryce was or was not mistaken in this case. Yet it does appear that Dr. Bryce was covertly and severely assaulted by some unknown party in a manner calculated to excuse, in some measure, the severity of his retort, while the testimonials of Drs. Manson and McGuire show that the charges made against him were not sustained by the facts.

